# Three-Dimensional Printing of Continuous Flax Fiber-Reinforced Thermoplastic Composites by Five-Axis Machine

**DOI:** 10.3390/ma13071678

**Published:** 2020-04-03

**Authors:** Haiguang Zhang, Di Liu, Tinglong Huang, Qingxi Hu, Herfried Lammer

**Affiliations:** 1 Rapid Manufacturing Engineering Center, School of Mechatronical Engineering and Automation, Shanghai University, Shanghai 200444, China; ld523257293@shu.edu.cn (D.L.); tlhuang@shu.edu.cn (T.H.); 2 Kompetenzzentrum Holz GmbH, Altenberger Straße 69, 4040 Linz, Austria; h.lammer@wood-kplus.at

**Keywords:** 3D printing, fused filament fabrication, continuous fiber-reinforced, five-axis 3D printer

## Abstract

A method for printing continuous flax fiber-reinforced plastic (CFFRP) composite parts by five-axis three-dimensional (3D) printer, based on fused filament fabrication (FFF) technology, has been developed. FFF printed parts usually need supporting structures, have a stair step effect, and unfavorable mechanical properties. In order to address these deficiencies, continuous natural fiber prepreg filaments were first manufactured, followed by curved path planning for the model for generation of the G-code, and finally printed by a five-axis 3D printer. The surface quality of printed parts was greatly improved. The tensile strength and modulus of CFFRP increased by 89% and 73%, respectively, compared with polylactic acid (PLA) filaments. The flexural strength and modulus of the 3D-printed CFFRP specimens increased by 211% and 224%, respectively, compared with PLA specimens. The maximal curved bending force load and stiffness of the 3D-printed CFFRP specimens increased by 39% and 115%, respectively, compared with the flat slicing method. Advanced light structures, such as leaf springs, can be designed and manufactured by taking advantage of the favorable properties of these composites, which endow them with significant potential for application in the field of automobiles.

## 1. Introduction

Three-dimensional (3D) printing technology, also called rapid prototyping or additive manufacturing, has been rapidly developed and widely used in various fields [1]. Fused filament fabrication (FFF) is one of most widely used 3D printing technologies due to inexpensive equipment and materials, and ease of operation [2]. FFF forms a 3D geometry by slicing a model along the XY-plane and assembling the resulting individual layers along the Z-axis, with extruded thermoplastic filaments, such as acrylonitrile butadiene styrene (ABS), polylactic acid (PLA), polypropylene (PP), or polyethylene (PE) [3]. Much research has focused on the limitations of the FFF process, such as the stair step effect and inadequate mechanical strength, and put forward corresponding solutions.

In terms of process parameter optimization, Chacón et al. [4] characterized the effect of build orientation, layer thickness, and feed rate on the mechanical performance of PLA specimens. Alaimo et al. [5] studied the influence of meso-structure and chemical composition on the mechanical behavior of ABS specimens. Mohamed et al. [6] investigated the effect of the parameters on the viscoelastic response. Sood et al. [7] predicted and optimized the parameters using an artificial neural network (ANN) in conjunction with a bacterial-foraging optimization algorithm. Bayraktar et al. [8] also used ANN to analyze the impact of melt temperature, layer thickness, and raster pattern on tensile strength. In summary, melt temperature and rate speed have little effect on strength. The number of contours should be as large as possible, and the layer thickness should be as small as possible. In addition, 45° of the raster angle and 100% infill are the best.

Regarding path planning and the printer, Huang et al. [9,10,11], Jin et al. [12], Zhang et al. [13], and Allen et al. [14] proposed a curved layer slicing method in contrast with the flat layer slicing method. In order to print the path by curved layer slicing, it should be combined with a multi-axis printer. The printers are now available in many advanced design types. One such design is the Delta-type, whose variant forms include three pairs of moving parallel arms that hold the print-head in place during 3D printing operation. A more popular design is the Cartesian gantry design that is usually composed of drives along the X and Y axes, and a moving platform along Z. Isa et al. [15] added two degrees of freedom to the platform on the Delta printer model. Asif et al. [16] added the A and B axes to the extruder on the Cartesian printer model.

The main limitation of FFF is its inferiority in terms of the mechanical properties of the resulting prototypes. This led to the development of various types of alternative materials in order to improve the application domain of this technology [17]. Research on fiber-reinforced thermoplastic composites can be categorized according to the type or size of reinforcements [18]. The variables commonly used in experiments with short fiber-reinforced composites are weight fraction and fiber type, such as carbon fiber and glass fiber. Tekinalp et al. [19], Ning et al. [20,21], Anwer et al. [22], Jaszkiewicz et al. [23], and Ferreira et al. [24] studied different aspects of the above. In experiments with continuous fiber-reinforced composites, Tian et al. [25,26,27], Yao et al. [28], Zhang et al. [29], and Hao et al. [30] studied composite behavior and printing using continuous carbon fiber-reinforced plastic (CCFRP). On this basis, Hu et al. [31] manufactured continuous carbon fiber prepreg filaments, in which the flexural strength of the final printed parts can reach as much as 610 MPa. Caminero et al. [32] printed carbon, glass, and Kevlar fibers in the middle layer of thermoplastic using two extruders. Matsuzaki et al. [33] printed carbon and natural jute fiber composites by in-nozzle impregnation. In conclusion, continuous fiber-reinforced plastic fabricated using prepreg showed superior mechanical properties. 

However, many limitations persist in the CCFRP printing process due to the stiffness of carbon fiber; most machines adopt the solution of double nozzles (e.g., Markforged [34], Anisoprint [35]). In addition, the high-cost, high-energy consumption carbon fiber manufacturing process is still significant. In contrast, natural fibers have the characteristics of light density, low price, and abundant sources. If it is possible to replace carbon fibers with natural fibers in the field of light-weight manufacturing, it will be very beneficial. At the same time, in order to improve the printing quality and mechanical strength of composites, two degrees of freedom were added to the printer. Taking into consideration the factors discussed above, this paper presents a novel method to print continuous flax fiber-reinforced plastic (CFFRP). 

## 2. Materials and Methods

### 2.1. CFFRP Filament Manufacturing

**Figure 1:**
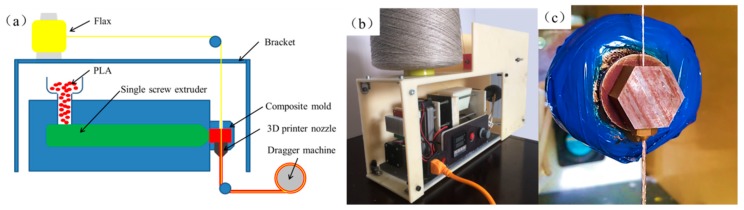
The production of 3D-printable continuous flax fiber-reinforced plastic (CFFRP) prepreg filaments. (**a**) Schematic for manufacturing CFFRP prepreg filaments. (**b**) Device for manufacturing CFFRP prepreg filaments. (**c**) Composite mold of device.

As shown in Figure 1, a single screw extruder and composite extrusion mold were employed for the manufacturing process. The process could be divided into four steps as follows: 

Heating: the diameter of PLA-GH401 (Guanghe Co., Ltd., Shanghai, China) pellets was 0.5 mm, added through the barrel, and the extruder was equipped with heating and heat preservation devices, so that the solid pellets were heated to a melting state and had certain fluidity. The heating band, which could be heated up to 300 °C, was placed in the front part of the single screw extruder. The melt extrusion temperature of PLA was set at 190 °C.

Extruding: the single screw extruder squeezed the molten PLA resin into the composite extrusion die. The extrusion speed of the screw was from 200 to 600 mm/min. The selected extrusion speed needed to be compatible with the composite filament diameter and collecting speed.

Compounding: the die had a vertical opening where a 3D printer nozzle was attached and the diameter of the upper hole was 1.2 mm. Flax fibers of which the diameter was about 0.5 mm were introduced and coated by the molten PLA resin as shown in Figure 1c. The diameter of the CFFRP prepreg filaments could be changed by varying the 3D printer nozzle. The flax was a two-ply yarn of 68Tex. The diameter of CFFRP prepreg filaments was set to 0.8, 1.0, and 1.2 mm by the 3D printer nozzle.

Collecting: the dragger machine maintained constant speed and tension during the entire manufacturing process. At the same time, the composite material was mechanically collected into rolls. 

### 2.2. 3D Printer

**Figure 2:**
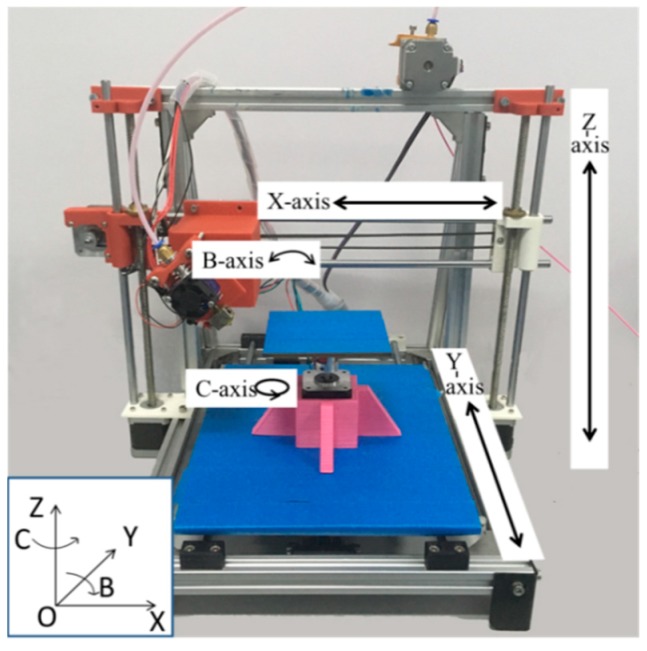
Five-axis 3D printer.

3D printed CFFRP specimens were manufactured using a five-axis 3D printer, which had an added B-axis with an extruder that rotated around the Y-axis and C-axis with a platform that rotated around the Z-axis, in addition to the three ordinary axes of the Cartesian printer model. As shown in Figure 2, the overall size of the printer frame was 470 × 450 × 460 mm, and the maximum size that could be printed was 100 × 100 × 100 mm. The C-axis platform and rotating mechanisms could be removed and therefore, without the C-axis, the printing size was 190 × 170 × 150 mm. When the B-axis pointed to the negative direction of the X-axis, it was 0°. However, it could be rotated from 0° to 180°, while the C-axis could be rotated freely (360°). The shortest line of 0.2 mm could be printed to approximate the curve. The printer’s extruder was modified, since the CFFRP could not be squeezed like pure melted PLA. The diameter of the nozzle matched the diameter of the CFFRP filaments. The printer adopted was the Atmega2560 (Airuide 3D Co., Ltd., Shenzhen, China), which used the same CPU, the Arduino MEGA, as the master control chip of the slave computer, called the RUMBA (RepRap Universal Mega Board with Allegro driver). Marlin 1.0.2 [36] was used as firmware to communicate with the host computer in order to control five-axis printing.

### 2.3. Tests

**Figure 3:**
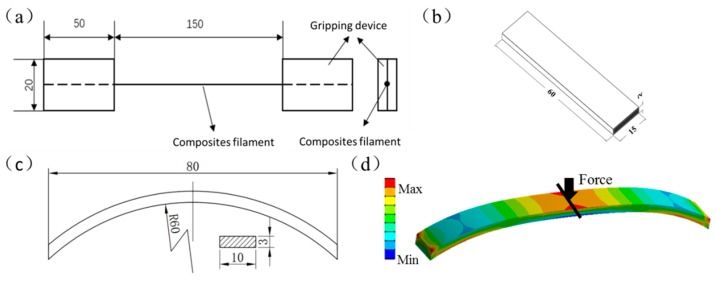
Standard specimens for testing. Dimensions are in mm. (**a**) Specimens for CFFRP tensile test. (**b**) Specimens for three-point bending test. (**c**) Specimens for curved bending test. (**d**) Analysis of force of curved structure.

Tensile, three-point bending and curved bending tests were conducted using the microcomputer controlled electronic testing machine (WDW-1, SONGDUN Corp., Shanghai, China). Surfaces of the filaments and fracture surfaces of the tested specimens were observed with a HITACHI SU-1510 (HITACHI Ltd., Tokyo, Japan) scanning electron microscope (SEM). The geometry of the specimens was designed using computer aided design software and exported as STL files. The main dimensions of the specimens are shown in Figure 3. For tensile tests of filaments (refer to ASTM D4018 [37]), test length was 150 mm (Figure 3a). The tensile strength was $\;\sigma_{t}{}_{\;}$ using $\;\sigma_{t}={\frac{F}{A}}_{\;}$, where *F* is the tensile load and *A* is the cross-sectional area of the specimen. The tensile modulus was $\;E_{t}{}_{\;}$ using $\;E_{t}={\frac{\Delta S}{\Delta S_{t}}}_{\;}$, where ∆*S* is the stress increment and ∆*S_t_* is the strain increment.

For three bending tests of CFFRP-printing (refer to ISO 14125:1998 [38]), the specimens were 60 mm × 15 mm × 2 mm, and the support span was 40 mm (Figure 3b). The flexural strength was $\sigma_{f}\;$using$\;\sigma_{f}={\frac{3F\times L}{2b\times h^{2}}}_{\;}$, where *F* is the failure load (N), l is the distance between the two supports, *b* is the width of specimen, and *h* is the thickness of the specimen. The *L* was set at 40 mm. The flexural modulus was$\;E_{f}$ using $\;E_{f}={\frac{L^{3}\times \Delta P}{4b\times h^{3}\times \Delta S}}_{\;}$, where *L*, *b,* and *h* have the same meaning as the variables above; ΔP is the increment of load in the initial linear part of the load and deflection curve; and ∆*S* is the increment of deflection corresponding to ΔP at the midpoint of *L*.

**Figure 4:**
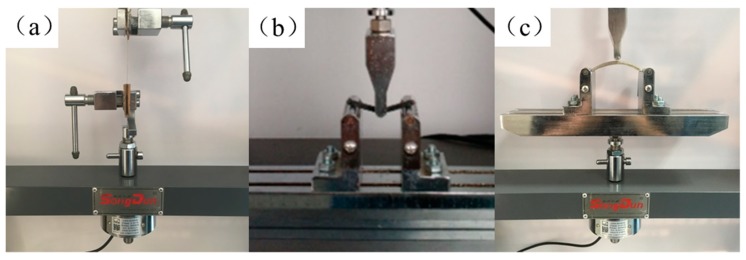
Specimens and tests. (**a**) Tensile test of CFFRP prepreg filaments. (**b**) Three-point bending test of continuous carbon fiber-reinforced plastic (CCFRP) specimens. (**c**) Curved bending test of curved CFFRP 1.0 mm specimens.

For curved bending tests, the specimens were 80 mm in length, 10 mm in width, 3 mm in thickness, and the radius of the curve was 60 mm (Figure 3c). When there was a load on the top of the specimen, analysis of force of the curved structure was calculated as shown in Figure 3d. It is known that the mechanical strength of fiber-reinforced polymers is highest in the direction of fiber alignment, so we tried to ensure that continuous fibers were arranged along the direction of the force gradient. The build orientation was divided into three types: upright, flat, and curved. The curved bending stiffness *k* (N/mm), which refers to the ability of structures to resist elastic deformation when subjected to a force, was calculated using$\;k=\frac{F}{\delta }\;$, where *F* is the force load (N) acting on the structure, and *δ* is the deformation (mm) due to the force. Tensile tests of the CFFRP prepreg filaments and bending tests of the specimens are shown in Figure 4.

In curved bending test experiments, all parameters of printing except the build orientation were constant. For PLA printing, the temperature of the nozzle was 200 °C, layer thickness was the height of a single layer, chosen as 0.35 mm, both the number of contours and number of roof and floor layers were 1, and the fill density of specimens was 100%. For upright and flat build orientation, the model was sliced using the 3D print Cura 15.04.6 [39] slicing engine, and the raster angle was 45°. For CFFRP printing, the thickness was 0.5 mm and the printing speed was 100 mm/min.

## 3. Results and Discussion

### 3.1. CFFRP Filament Tests

**Figure 5:**
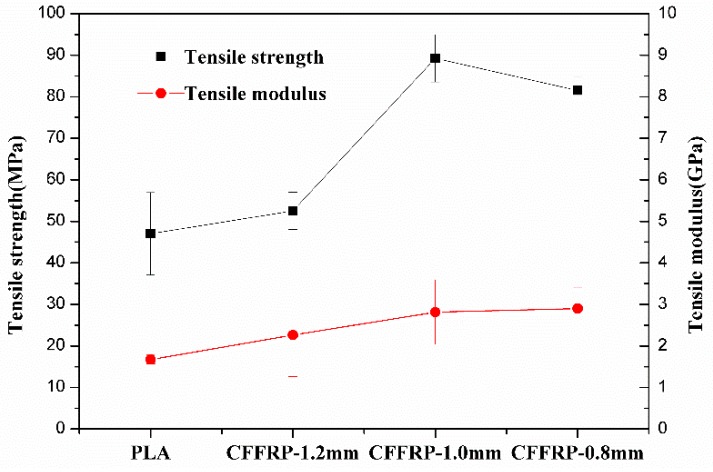
Tensile strength and modulus test results of CFFRP filaments.

**Figure 6:**
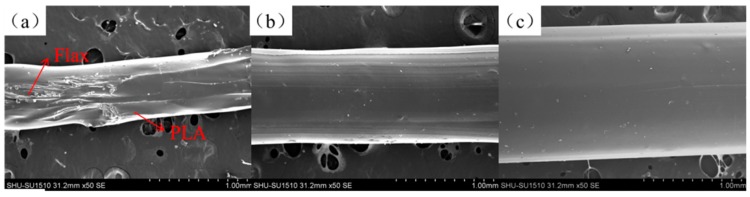
SEM images of the filament surfaces. (**a**) CFFRP 0.8 mm. (**b**) CFFRP 1.0 mm. (**c**) CFFRP 1.2 mm.

Tensile strength and tensile modulus test results of CFFRP filaments are shown in Figure 5. The tensile strength of PLA filaments was 47 MPa, and the tensile modulus was 1.7 GPa. When continuous flax fibers were coated by PLA, the tensile strength increased with the decrease of the CFFRP composite’s diameter, which meant that the proportion of flax fiber increased. The weight fraction of flax in CFFRP is depicted in Table 1. The maximum tensile strength was 89 MPa when the diameter of the CFFRP was 1.0 mm, and the maximum tensile modulus was 2.9 GPa when the diameter of the CFFRP was 0.8 mm. SEM images of the filament surfaces are shown in Figure 6. The reason for the slight decrease in the tensile strength when the CFFRP diameter was 0.8 mm was that the surface-coated PLA could not be uniformly wrapped around the flax fiber due to the limitations of the machine, and there would be thinner or uncoated sections of the fiber; but a diameter of 1.0 mm would avoid this problem. In addition, because the composite mold had a vertical opening, during the process of composite materials forming, air would enter from the top of the opening, resulting in bubbles in the composite material and slightly increased surface roughness of the filaments. However, this did not affect the printing because the PLA outside the composite material was re-melted during the printing process.

### 3.2. Three-Point Bending Test of CFFRP

**Figure 7:**
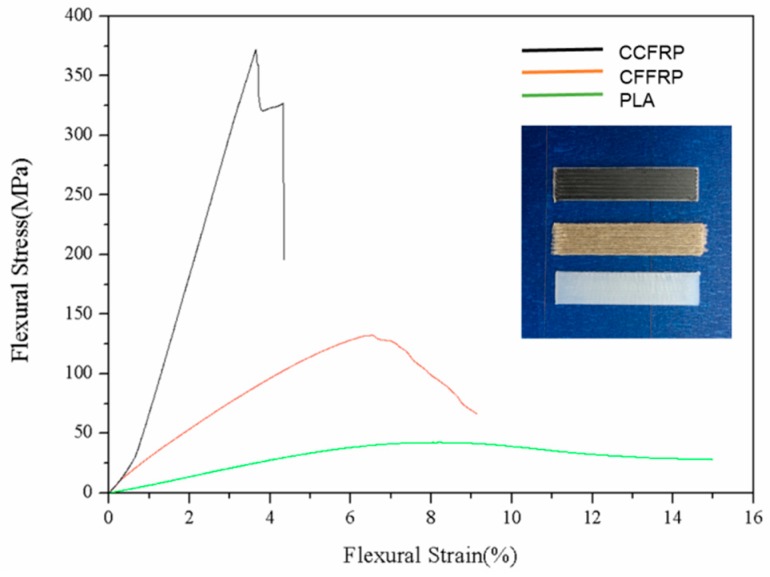
The three-point bending test of CCFRP, CFFRP, and PLA.

Figure 7 displays the three-point bending test results of three specimens printed by CCFRP, CFFRP, and PLA. Flexural strength of CFFRP-printed specimens was 132 MPa, 211% higher than PLA-printed, which had flexural strength of 42 MPa, but much lower than CCFRP-printed, which had flexural strength of 372 MPa. Flexural modulus of CFFRP-printed specimens was 7.0 GPa, 224% higher than PLA-printed, which had flexural modulus of 2.2 GPa. The flexural modulus of CCFRP-printed was 34.9 GPa. Although a reinforcing effect like that of CCFRP was not achieved, comprehensive consideration of the cost, environmental protection, and degradability of natural fibers suggests CFFRP is a reinforcing material with great application potential.

### 3.3. Build Orientation of Specimens

**Figure 8:**
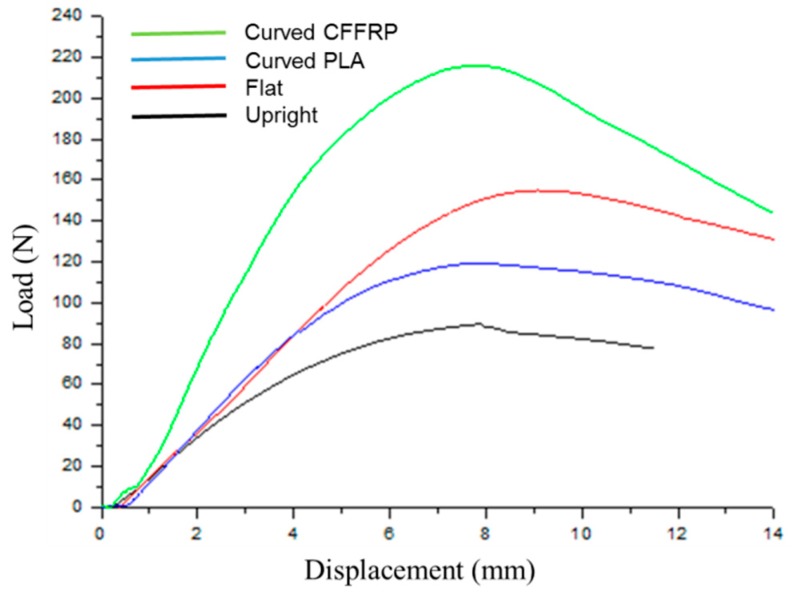
Load-displacement curves of curved bending test.

**Figure 9:**
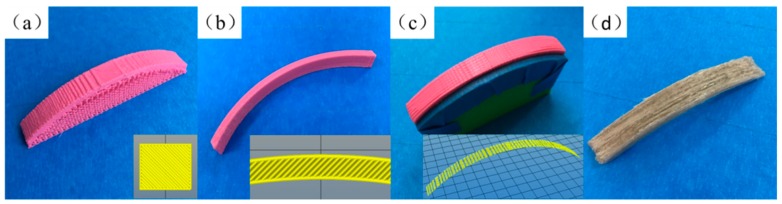
The build orientation of specimens. (**a**) Upright. (**b**) Flat. (**c**) Curved. (**d**) Curved CFFRP 1.0 mm.

**Figure 10:**
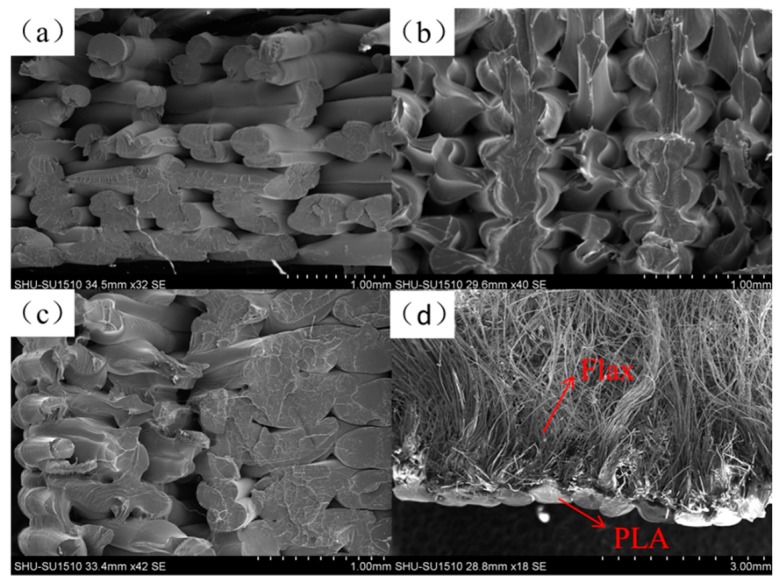
SEM images of the fractured surfaces of PLA specimens. (**a**) Upright. (**b**) Flat. (**c**) Curved. (**d**) Curved CFFRP.

Figure 8 depicts load-displacement curves of the curved bending test. As shown in Figure 9a, when the build orientation was upright, the stair step effect was obvious. The maximal curved bending load was 90 N and the stiffness was 14.8 N/mm. In the slicing software, the support density and distance between the support and the model entity were adjustable. However, the less the support and the larger the distance, the easier it was for the support to peel off, leading to poor support of the model and model deformation. A more serious problem was that during the process of support stripping, the surface of the model would be damaged, and the roughness would be greatly increased. In order to solve this problem, the method of printing water-soluble support materials with dual nozzles has been proposed, but it increased the cost of printing and forfeited the advantages of the FFF process. Compared with Figure 9b, when the build orientation was flat, it needed no support. However, it was not applicable to all curved surface models. Most models still needed to be supported when they were printed in the flat direction, and the surface roughness was similar to that obtained when the direction was upright. For the maximum curved bending load shown in the samples printed in PLA (non-reinforced), the value was 155 N because the 45° intersection filling path provided a good load-bearing capacity on the stress surface in the curved bending test. Furthermore, there were large voids printed when the build orientation was upright or flat as shown in Figure 10a,b. Due to the limitation of the slicing algorithm and the shape of the extruded filaments, voids were inevitable, but reducing the voids to obtain a larger filling rate could improve the mechanical properties.

Figure 9c displays the specimens of the curved build orientation by the five-axis machine. Although the specimens still needed support, the support did not peel off so that they were destroyed, and could be reused with the help of masking tape. The curved path eliminated the stair step effect and improved the surface quality. In the curved bending test, the maximal curved bending load was 119 N, slightly lower than that in flat printing, which was due to the compactness of filling; that is, the void formation inside the model. The motor that controlled the rotation of the extruder was set on the X-axis rail. The additional weight of the motor caused the extruder to tilt downward slightly, which increased the instability of the nozzle. Furthermore, excessive printing speed and uneven support could cause a slight jitter of the extruder. Therefore, existing 3D printers mostly use double-rod cross-axes to install and fix, because the more stable the extruder, the higher the accuracy; the more compact the filling, the more favorable the mechanical properties. Moreover, the stiffness was the highest in PLA specimens, and the value was 23 N/mm. Because the curved path ensured the continuity of the filling of the filaments, the deformation of the continuous filaments required greater force (Figure 9c).

Figure 9d and Figure 10d show the details of the CFFRP specimens including fractured surfaces. Maximal curved bending load and stiffness of CFFRP composite materials were 39% and 115% higher respectively than those of flat build orientation, and 81% and 88% higher respectively than those of the same curved path printing of PLA filaments. Due to reinforcement by the flax fibers, the performance of the specimens was significantly improved.

**Figure 11:**
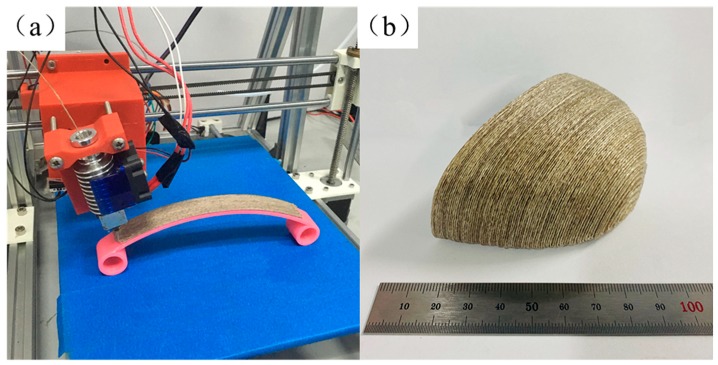
CFFRP component fabricated by five-axis 3D printing machine. (**a**) Leaf spring; (**b**) Shoe cap.

A 3D printed leaf spring part was fabricated using CFFRP prepreg filaments and curved path planning as shown in Figure 11a. The leaf spring bears the impact of the wheel on the frame, reduces the violent vibration of the body, and maintains the stability of the vehicle, and the adaptability to different road conditions. When the leaf spring was subjected to load shock, it underwent a stretching motion; that is, extrusion tension. An advanced structure such as a shoe cap (Figure 11b) can be designed and manufactured by taking advantage of the favorable properties of this composite, which endows it with significant potential for application. The average density of specimens printed by CFFRP 1.0 mm composites was around 1.167 g/cm^3^.

## 4. Conclusions

In order to improve the FFF process, prepreg filaments were manufactured as a first step. Subsequently, the angle of the extruder was calculated according to the path of the curved layer. Finally, the specimens were printed by a five-axis 3D printer which had a B-axis added to the extruder and a C-axis added to the platform of the Cartesian printer model. We studied the results of tensile tests of CFFRP and curved bending tests of printed specimens. Different ranges of two main process parameters were analyzed: the diameter of filaments and build orientation. Both achieved considerable improvement compared to parts printed with pure PLA. CFFRP filaments and curved path printing make up for the deficiencies of the FFF process. If natural fibres such as flax can be used in FFF printing of functional parts, it will have far-reaching significance in the field of composite fabrication where most demand is for low-weight and high-performance parts.

## Figures and Tables

**Table 1:** Weight fraction of flax in CFFRP.

Diameter of CFFRP (mm)	Weight Fraction of Flax (%)
1.2	10.6
1.0	20.4
0.8	36.7
